# Supplementary material for: Microbial Dynamics during labneh Ambaris Production in Earthenware Jars
Source: Foods. 2023 Aug 21;12(16):3131. doi: 10.3390/foods12163131 (PMC10453334; doi:10.3390/foods12163131)
Supplement: Supplementary file 1 [file foods-12-03131-s001.zip › Figure S1.pdf]

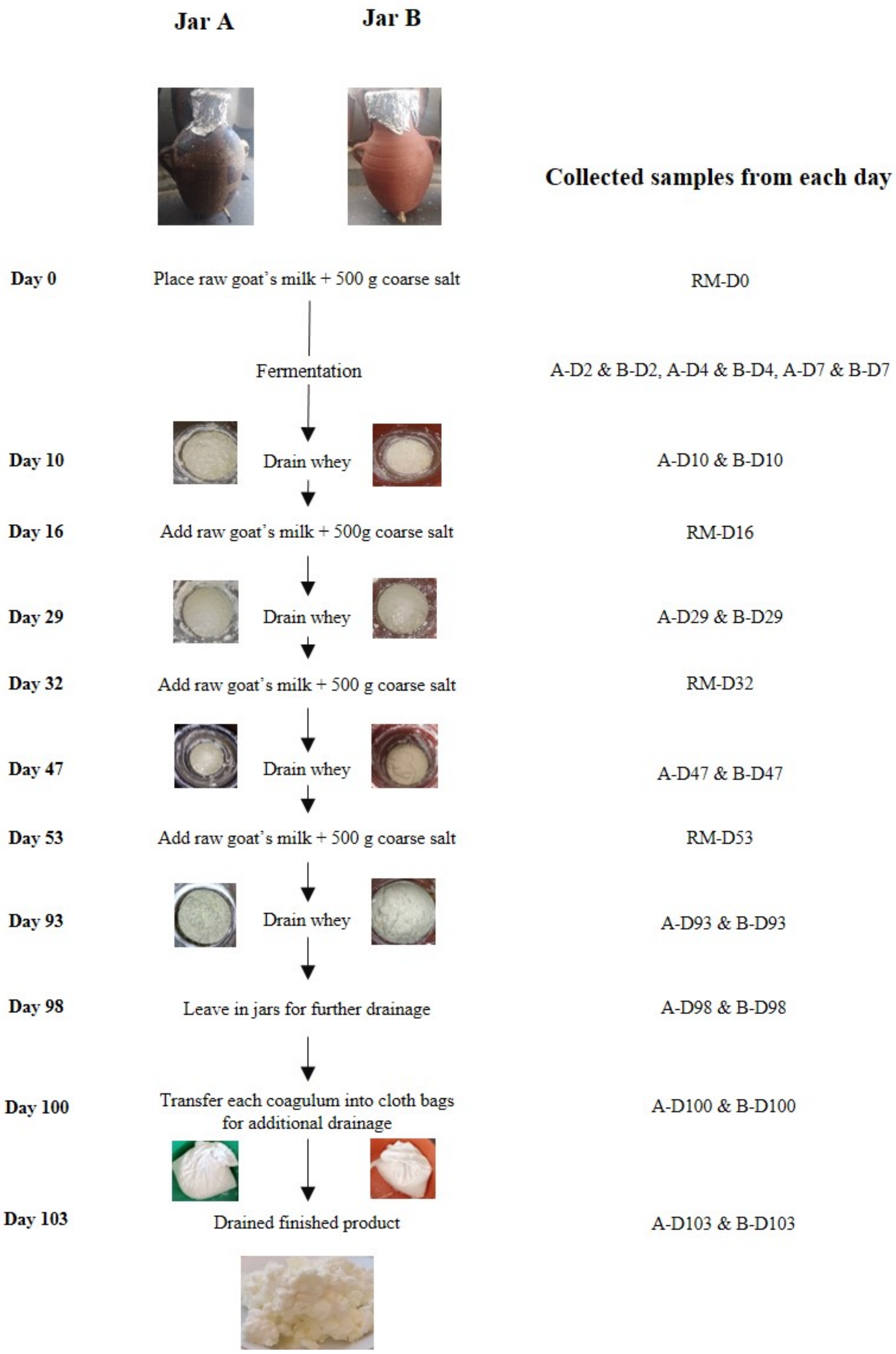

Figure S1. Detailed production process of two labneh Ambaris productions made earthenware jars along with the collected samples resulting from each step. RM codes followed by production days refer to raw milks added to both jars during productions. The fermented milks collected from the Jar A and the Jar B are coded as A or B respectively followed by the corresponding sampling day of production.
