# Supplementary material for: Microbial Dynamics during labneh Ambaris Production in Earthenware Jars
Source: Foods. 2023 Aug 21;12(16):3131. doi: 10.3390/foods12163131 (PMC10453334; doi:10.3390/foods12163131)
Supplement: Supplementary file 1 [file foods-12-03131-s001.zip › Figure S2.pdf]

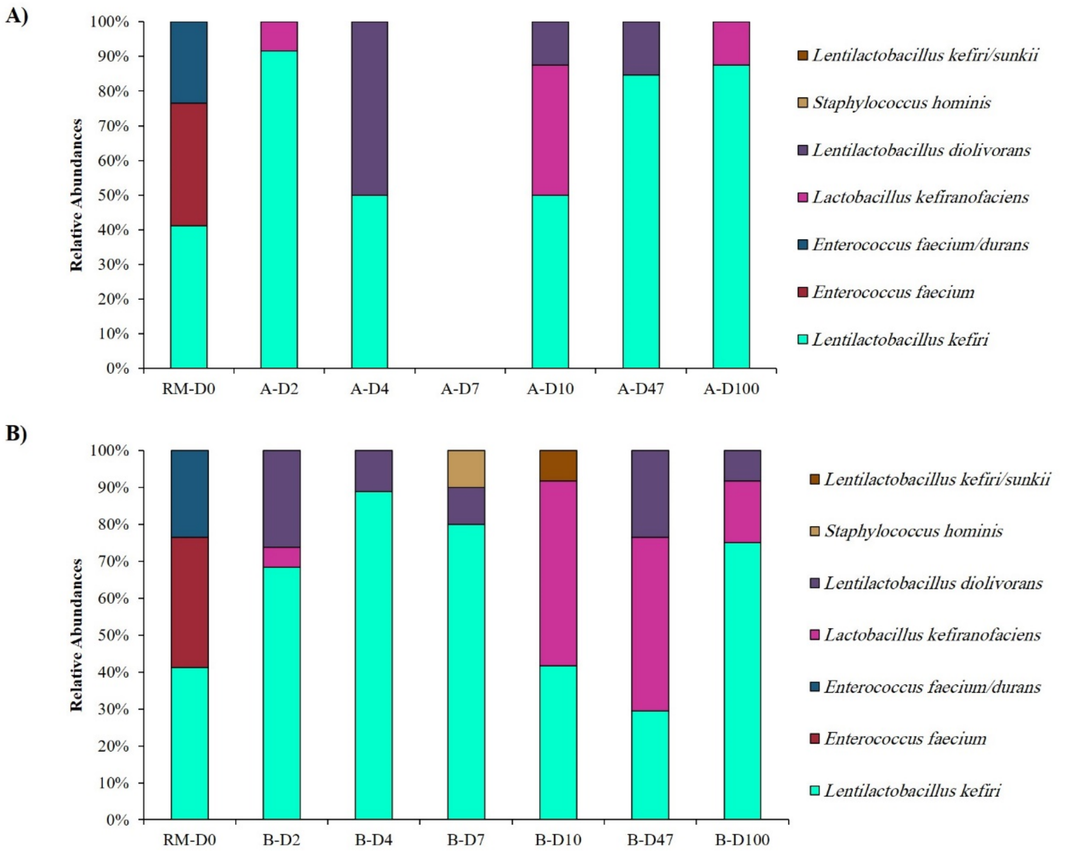

Figure S2. Bacterial community dynamics in labneh Ambaris samples collected during production from 2 earthenware jars, Jar A and Jar B, in addition to the initial raw milk by sequencing of the V1-V4 region of the 16S rRNA gene.
