# Supplementary material for: Microbial Dynamics during labneh Ambaris Production in Earthenware Jars
Source: Foods. 2023 Aug 21;12(16):3131. doi: 10.3390/foods12163131 (PMC10453334; doi:10.3390/foods12163131)
Supplement: Supplementary file 1 [file foods-12-03131-s001.zip › Table S1.pdf]

Table S1. Microbial counts obtained on 5 nutritive and selective media during production of labneh Ambaris from D0 until the final products. Two productions were monitored in two earthenware jars (Jar A and Jar B). ND means not determined. The values presented here are the 2 values (n=2) for each sample and the average value.

Sampling day	Sample codes	Total mesophilic aerobic flora Value 1 / Value 2 (log cfu/g or mL)	Average total mesophilic aerobic flora (log cfu/g or mL)	Total presumptive lactobacilli Value 1 / Value 2 (log cfu/g or mL)	Average total presumptive lactobacilli (log cfu/g or mL)
D0	RM-D0	6.30 / 6.21	6.26	4.46 / 4.47	4.46
D2	A-D2	8.78 / 8.79	8.79	8.11 / 8.08	8.10
	B-D2	ND / 5.83	5.83	8.11 / 8.23	8.18
D4	A-D4	6.70 / 6.78	6.74	ND / 6.31	6.31
	B-D4	7.80 / 8.23	8.00	8.05 / 7.74	7.90
D7	A-D7	6.25 / 6.34	6.30	8.40 / 8.41	8.40
	B-D7	8.00 / 7.96	7.98	9.11 / 8.62	8.87
D10	A-D10	7.91 / 8.09	8.01	8.18 / 8.12	8.16
	B-D10	8.09 / 8.12	8.10	8.26 / 8.31	8.28
D29	A-D29	8.20 / 8.25	8.23	7.53 / ND	7.53
	B-D29	7.04 / 7.13	7.09	ND / 8.35	8.35
D47	A-D47	6.48 / 6.85	6.66	6.48 / 6.20	6.34
	B-D47	7.38 / ND	7.38	7.27 / 7.08	7.18
D93	A-D93	7.56 / 7.54	7.55	7.36 / 7.24	7.31
	B-D93	7.66 / 7.46	7.57	ND / 7.58	7.58
D98	A-D98	7.73 / ND	7.73	ND / ND	ND
	B-D98	7.96 / ND	7.96	ND / ND	ND
D103	A-D103	5.34 / ND	5.34	4.34 / ND	4.34
	B-D103	5.64 / ND	5.64	5.11 / ND	5.11

Sampling day	Sample codes	Total yeasts and molds Value 1 / Value 2 (log cfu/g or mL)		Average total yeasts and molds (log cfu/g or mL)	Total *Enterobacteriaceae* family Value 1 / Value 2 (log cfu/g or mL)	Average total *Enterobacteriaceae* family (log cfu/g or mL)	Total coliforms Value 1 / Value 2 (log cfu/g or mL)	Average total coliforms (log cfu/g or mL)
D0	RM-D0	5.13	5.06	5.10	4.76 / 4.69	4.72	4.78 / 4.86	4.82
D2	A-D2	ND	6.32	6.32	6.47 / 6.43	6.45	5.58 / 5.56	6.57
	B-D2	6.43	6.44	6.44	4.40 / 4.30	4.35	5.48 / 5.54	4.51
D4	A-D4	6.85	6.97	6.91	3.18 / 3.04	3.11	<10 / <10	<10
	B-D4	7.04	7.25	7.16	4.54 / 4.60	4.57	4.44 / 4.34	4.39
D7	A-D7	5.34	ND	5.34	<10 / <10	<10	<10 / <10	<10
	B-D7	8.40	8.39	8.39	<10 / <10	<10	<10 / <10	<10
					<10 / <10	<10	<10 / <10	<10
D10	A-D10	8.11	8.11	8.11	<10 / <10	<10	<10 / <10	<10
	B-D10	8.42	8.05	8.24	<10 / <10	<10	<10 / <10	<10
					<10 / <10	<10	<10 / <10	<10
D29	A-D29	8.17	8.19	8.18	<10 / <10	<10	<10 / <10	<10
	B-D29	6.11	6.15	6.13	<10 / <10	<10	<10 / <10	<10
					<10 / <10	<10	<10 / <10	<10
D47	A-D47	ND	6.37	6.37	<10 / <10	<10	<10 / <10	<10
	B-D47	7.75	7.77	7.76	<10 / <10	<10	<10 / <10	<10
					<10 / <10	<10	<10 / <10	<10
D93	A-D93	6.72	6.30	6.51	<10 / <10	<10	<10 / <10	<10
	B-D93	6.40	6.44	6.42	<10 / <10	<10	<10 / <10	<10
					<10 / <10	<10	<10 / <10	<10
D98	A-D98	4.96	ND	4.96	<10 / <10	<10	<10 / <10	<10
	B-D98	3.48	ND	3.48	<10 / <10	<10	<10 / <10	<10
					<10 / <10	<10	<10 / <10	<10
D103	A-D103	3.27	ND	3.27	<10 / <10	<10	<10 / <10	<10
	B-D103	2.64	ND	2.64	<10 / <10	<10	<10 / <10	<10
