# Supplementary material for: Microbial Dynamics during labneh Ambaris Production in Earthenware Jars
Source: Foods. 2023 Aug 21;12(16):3131. doi: 10.3390/foods12163131 (PMC10453334; doi:10.3390/foods12163131)

Table S2. Microbial counts obtained on lwMRS media during the production of labneh Ambaris at different time points throughout fermentation in 2 earthenware jars

	Microbial counts (log cfu/g or mL)	
	Jar A	Jar B
D0	6.39	6.39
D2	6.15	6.5
D4	5.95	6.16
D7	0	4.1
D10	4.36	4.44
D47	7.36	7.66
D100	8.35	8.13

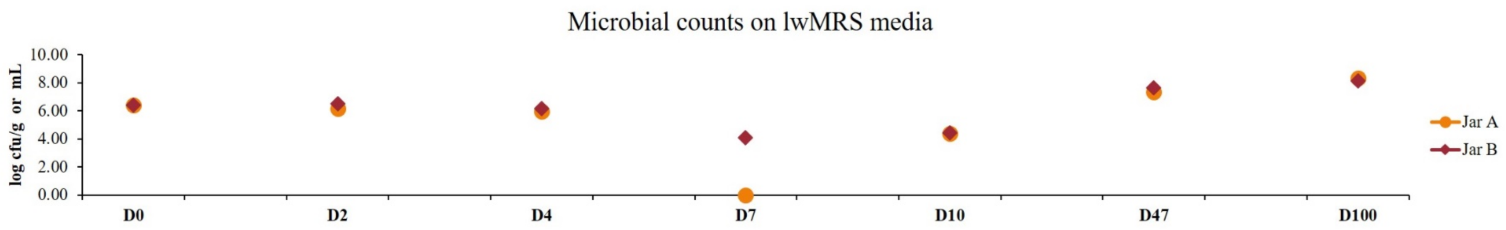

Supplement: Supplementary file 1 [file foods-12-03131-s001.zip › Table S2.pdf]
